# Serum Metabolomic Profiling Reveals the Amelioration Effect of Methotrexate on Imiquimod-Induced Psoriasis in Mouse

**DOI:** 10.3389/fphar.2020.558629

**Published:** 2020-11-19

**Authors:** Jiaxin Zong, Jieyi Cheng, Yuanfeng Fu, Jing Song, Weisong Pan, Li Yang, Ting Zhang, Mingmei Zhou

**Affiliations:** ^1^ Murad Research Center for Modernized Chinese Medicine, Institute for Interdisciplinary Medicine Sciences, Shanghai University of Traditional Chinese Medicine, Shanghai, China; ^2^ Center for Chinese Medicine Therapy and Systems Biology, Institute for Interdisciplinary Medicine Sciences, Shanghai University of Traditional Chinese Medicine, Shanghai, China; ^3^ School of Pharmacy, Shanghai University of Traditional Chinese Medicine, Shanghai, China; ^4^ Guangzhou Institute for Drug Control, Guangzhou, China

**Keywords:** methotrexate, imiquimod, psoriasis, metabolomics, gas chromatography-mass spectrometry

## Abstract

**Background:**

The imiquimod (IMQ)-induced psoriasis mouse model has been used as a model for pathogenic mechanism research, and methotrexate (MTX) is widely employed to treat various clinical manifestations of psoriasis. We explored the underlying pathogenesis of psoriasis and the treatment mechanism of the conventional drugs from the metabolic perspective of the psoriasis mouse model.

**Methods:**

Male BALB/c mice were smeared IMQ for 7 days to induce treatment-resistant psoriasis and intragastrically administered 1 mg/kg MTX. We evaluated inflammation of psoriasis-like lesions and therapeutic effects of MTX based on histological changes and immunohistochemistry. Based on gas chromatography-mass spectrometer detection of serum samples, a comprehensive metabolomics analysis was carried out to identify alterations of metabolites.

**Results:**

It was found that MTX ameliorated psoriatic lesions (representative erythema, scaling, and thickening) by inhibiting proliferation and differentiation of keratinocytes. Using multivariate statistical analysis to process metabolomics data, the results displayed alterations in serum metabolites among mice of the control group, IMQ group, and MTX group. Compared with group, psoriasis mice had the higher level of d-galactose and lower expression of myo-inositol, 9,12-octadecadienoic acid, and cholesterol. In contrast with the model set, serum levels of glycine, pyrrolidone carboxylic acid, d-galactose, and d-mannose were significantly decreased in the MTX group.

**Conclusion:**

The differential metabolites, reflecting the perturbation in the pathways of inositol phosphate metabolism; galactose metabolism; glyoxylate and dicarboxylate metabolism; glycine, serine, and threonine metabolism; and glutathione metabolism, may lead to the pathogenesis of psoriasis, and they are also related to the pharmacological treatment effect of MTX on psoriasis. This study established the foundation for further research on the mechanism and therapeutic targets of psoriasis.

## Introduction

Psoriasis is a chronic, palindromic, inflammatory dermatosis, and the proportion of symptomatic cases accounts for 2–4% of the population worldwide (Parisi et al., 2013). It is characterized phenotypically by skin erythema, thickening, and scaling formation and well established a link between psoriasis and obesity (Gladman et al., 2005; Carrascosa et al., 2014). The etiology of psoriasis has not been fully elucidated, but confirmed by the crosstalk between genetic and environmental factors which contribute to the dysregulation of keratinocyte differentiation that it demonstrates hyperproliferation, damage of the skin barrier, and immune dysfunction (Nickoloff et al., 2006; Hugh and Weinberg, 2018). The related report indicated that one of the pathogeneses of psoriasis is hypernomic activation of toll-like receptors in dendritic cells, which is basically believed to be crucial in initiating an immune response (Meng et al., 2017). To date, most studies have investigated feasible treatments for the disease, which may require the fundamental role for short-term improvement and long-term control, but there is no standardized therapeutic regimen for psoriasis and is incapable of resulting in the complete clearing of psoriasis (Al-Suwaidan and Feldman, 2000; Naldi et al., 2003; Krajewska-Wlodarczyk et al., 2018).

Methotrexate (MTX), the folic acid analog, is widely employed as therapy for inflammatory disorders including psoriasis, dermatomyositis, and lupus erythematous (Rajitha et al., 2017; Ye and Coates, 2019). Clinical mechanism study revealed that MTX suppressed the occurrence of inflammation in joint tissues by restraining the activation of NF-kB and NLRP3/caspase-1 pathways and adjusting the inflammation-linked metabolic networks (Pang et al., 2018). Meanwhile, it induced apoptosis of proliferating keratinocyte in psoriasis patients rapidly (Elango et al., 2017). Although newer biological agents are available for many patients with chronic inflammatory diseases, MTX is still a first-line drug for systemic treatment (Greb et al., 2016).

Animal models are extremely important experimental methods and means in modern biomedical research. Imiquimod (IMQ) is a ligand of TLR7/8, used as an effector for immune activation, and is closely related to the activation and maturation of dendritic cells (Dika et al., 2006; Stary et al., 2007). Evidence showed that the psoriasis mouse model induced by IMQ extremely resembles human psoriasis, and they are all related to the IL-23/IL-17 axis (van der Fits et al., 2009). It has been used as a model for the pathogenic mechanism research of psoriasis. In the experimental system, the IMQ-induced psoriatic itch has been implicated in the underlying mechanism of histaminergic and nonhistaminergic itch in psoriasis, as well as the sensibility of itch signal channels (Mahil et al., 2016). More and more emerging research reports indicated that IMQ-treated mice model has been widely used to investigate diseases related to inflammation and immunity, especially psoriasis (Chen et al., 2020; Da et al., 2020; Watanabe et al., 2020).

The importance of metabolites has been supported by the building blocks of cellular action. Since upstream biological dysregulation leads to a succession of metabolomic alteration, metabolomics, with a large amount of information discovered from metabolic research, has great values for predicting the phenotype (Schrimpe-Rutledge et al., 2016). These small-molecule metabolites involved a close interplay between genetic inheritance and diverse environmental stimulants. Over recent years, the incremental body of investigation has been made significant progress in the characterization of the underlying pathogenic mechanisms in psoriasis, and this phenomenon was more prevalent in genomics, proteomics, and transcriptomics research studies (Lu et al., 2014; Swindell et al., 2016; Kim et al., 2018). Another evidence further highlighted the importance of the metabolic byproducts, which may provide a basis to regulatory processes of cellular pathways and potential molecular networks at the transcriptional and translational levels. For more details, metabolites are the final products and the most downstream representation for cell metabolism. Therefore, through analyzing the amplified ultimate products caused by perturbations from gene and environmental factors, a comprehensive metabolomics research will be able to obtain critical physiological or pathological information of biological systems (Eckhart et al., 2012). In addition, the appropriate technologies have recently made great headway and allowed for providing novel insights into metabolomics for the elaboration of numerous broad-ranging fields in the pathogenic disease model which included toxicology, disease and drug mechanism of action, as well as new targets and biomarkers (Watson et al., 2009; Takei et al., 2010; Zgoda-Pols et al., 2011). Although relevant reviews have described the pathogenesis of psoriasis and the function of MTX in the therapy of psoriasis (Rajitha et al., 2017), the pathogenesis mechanism of IMQ-induced psoriasis in mice and the pharmacological principles of MTX for psoriasis have not been clarified completely yet with nontargeted metabolomics techniques.

In the present study, the amelioration effect of MTX on psoriatic lesions induced by IMQ was investigated by conventional Psoriasis Area Severity Index (PASI) scoring system, histology, and immunohistochemistry studies. Furthermore, a GC-MS-based untargeted metabolomics has been developed to reveal the serum level of differentiated metabolites in IMQ-induced psoriasis mice, as well as with MTX treatment. Representative alterations to differential metabolites and the perturbed pathways documented in this study established the foundation for further research on the mechanism and therapeutic targets of psoriasis and elucidated the amelioration effect of MTX on IMQ-induced psoriasis mouse model.

## Materials and Methods

### Psoriatic Model in Mice

BALB/c mice (male, 18–22 g, 8 weeks old) were purchased from Shanghai SIPPR-Bk Lab Animal Co., Ltd. (China) (certification no. SCXK 2018-0,006) and bred under specific conditions free of pathogens with free access to food and water in the Animal Experiment Center of Shanghai University of Traditional Chinese Medicine. Institutional animal ethics committee authorized all the experiments which were in accordance with the rules and regulations for animal experiments of Shanghai University of Traditional Chinese Medicine.

The experimental mice were divided into the following three groups, with eight mice in each group. Two groups received 42 mg daily dose of topical medication containing 5% IMQ (Mingxin Pharmaceutical, Sichuan, China) on the shaved back (2 × 3 cm) of each mouse to establish an IMQ-induced psoriasis mouse model (Qin et al., 2014). The control group was treated with appropriate vaseline. The model group was treated with pure water, while the MTX group recieved 1 mg/kg MTX (Shanghai Sine Pharmaceutical Laboratories Co., Ltd., Shanghai) (Menting et al., 2016). From the first day of IMQ-treatment, all treatments were operated once a day for 7 days. After 7 days of treatment, with anesthesia of sodium pentobarbital, mice were sacrificed by cervical dislocation. Skin lesions and serum samples were collected for further analysis.

### Scoring of Skin Inflammation Severity

The severity of skin lesions was investigated by a modified human scoring system, PASI (Meng et al., 2017). Thickening, scaling, and erythema were independently scored by the same experiment participant on 0–4 scales, that is, 0, none; 1, mild; 2, moderate; 3, marked; and 4, obvious. The trend lines were generated, and the accumulative score were calculated to denote severity of inflammation.

### Histology

The skin lesions were first fixed in 4% paraformaldehyde and embedded in paraffin and then slit 5 μm from the paraffin sections on a microtome. According to standard procedures, tissue slices (5 μm) were stained with hematoxylin and eosin (H&E) for pathological observation by light microscopy (Nikon Eclipse E100). Epidermal thickness, a recognized end-point for measuring the severity of psoriasis, was accurately measured with ImageJ software (National Institutes of Health, Bethesda, MD, United States).

### Immunohistochemistry

Anti-rabbit proliferating cell nuclear antigen (PCNA) (GB11010, 1:500, Wuhan Servicebio Technology Co., Ltd., Wuhan) was applied for immunostaining of skin samples of the back skin lesions, then staining was assessed, and photomicrographs were taken using a light microscope (Nikon Eclipse E100, Japan).

### Serum Sample Preparation for Metabolic Analysis

Blood was collected from the orbits after enucleation of the eyes, kept in room temperature for 1 h, then centrifuged for 10 min at 4,000 rpm, 4°C, and supernatants were gathered and immediately stored at −80°C for the subsequent test.

With modifications on our previous publication (Zhao et al., 2018), serum samples were conducted for GC-MS analysis to acquire corresponding spectral date. First, protein degradation was performed on each serum sample of 50 μl by adding 200 μl of methanol (25°C, 10 min). Then, the sample was derivatized with MSTFA (containing 1% TMCS) with heptadecanoic acid as the internal standard.

### Data Acquisition of the Full-Scan Sample

Serum data acquisition of the samples was analyzed using GC-MS (Pegasus HT, Leco Corp., St. Joseph, MI). Three blank vials and one QC sample were run after every eight analytical samples. A sample of 1 μl was injected into the DB-5MS capillary column (30 m × 250 μm i.d., 0.25 m film; Agilent, CA). High-purity helium gas with a constant flow rate of 1.0 ml/min was used as carrier gas. Temperature of the inlet was set to 260°C, while that of the quadropole and ion source was, set to 150 and 230°C, respectively. The oven temperature was started up at 80°C, held for 2 min, improved to 240°C at a rate of 5°C/min, followed by an increment of 25°C/min to 290°C, and finally kept for 10 min. The ionization mode was set with 70 eV electronic impact.

### Data Processing

The raw data from GC-MS analysis were pretreated by R 2.13.2 (Lucent Technologies), and the exported data containing peak area, retention time, and compounds name were employed to achieve principal component analysis (PCA) (He et al., 2009) and partial least squares discriminant analysis (PLS-DA) (He et al., 2011) by multivariate analysis with SIMCA-P 11 software (Umetrics, Umea, Sweden). Furthermore, the selection of differential metabolites was accomplished through orthogonal projection to latent structure with discriminant analysis (OPLS-DA) with variable importance in the projection (VIP) value >1.0. Additionally, potential biomarkers were identified using the commercial compound libraries, NIST, and screened out which were further verified by Kyoto Encyclopedia of Genes and Genomes (KEGG; http://www.genome.jp/kegg/) and Human Metabolome Database (HMDB; http://www.hmdb.ca/). The metabolic pathways of differential metabolites were confirmed with the metabolic pathway analysis module of MetaboAnalyst 3.0 (http://www.metaboanalyst.ca/).

SPSS 21.0 and two-tailed Student’s *t*-test were employed to analyze the data and implement statistical significance among the groups, respectively. All values were calculated as mean ± standard deviation (SD). A *p* value < 0.05 was set to show a statistically significant difference. Graphpad Prism 5.0 (GraphPad Software Inc., United States) was used to perform statistical analyses.

## Results

### Methotrexate Ameliorated Skin Lesions by Inhibiting Proliferation and Differentiation of Keratinocytes in the Psoriasis Mouse Model

**FIGURE 1:**
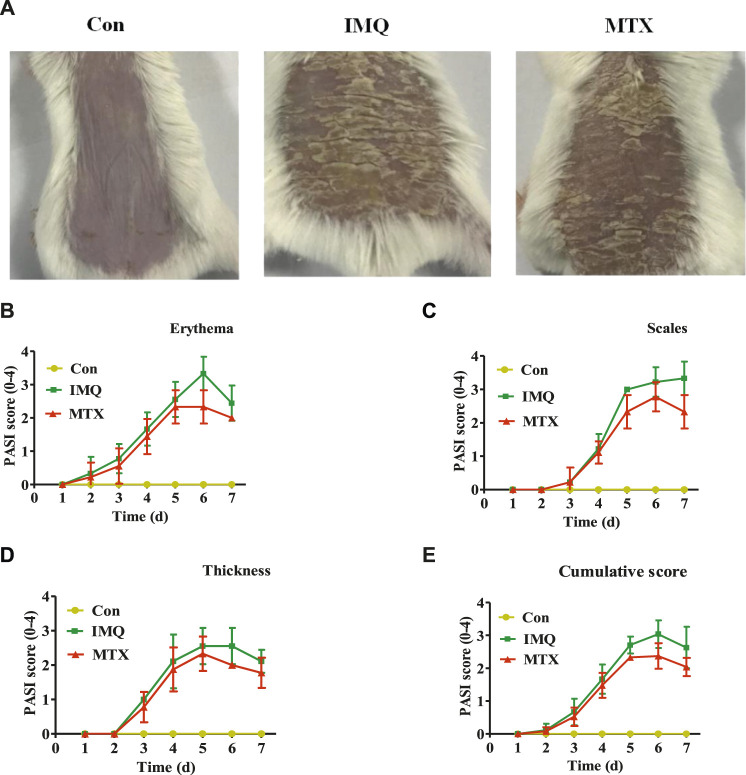
MTX treatment ameliorated IMQ-induced skin lesions. A mouse model of psoriasis was induced in mice by topical application of IMQ. Psoriasis-like skin lesions were monitored during 7 days in IMQ-treated animals. Administration of 1 mg/kg MTX exhibited ameliorated symptoms (data were represented as mean ± SD; *n* = 8). **(A)** Phenotypical presentation of the back skin in the different groups after IMQ exposure for 7 days. PASI score of erythema **(B)**, scales **(C)**, and thickness **(D)** on a scale from 0 to 4 and the cumulative score **(E)** were indicated.

Morphological observation was carried out to investigate the effect of MTX on the psoriatic mouse model caused by IMQ. Compared with the control group, IMQ-induced skin lesions displayed typical scaling, erythema, and thickening, while these pathological changes were significantly inhibited by MTX after 7 days of treatment (Figure 1A). No significant changes in the PASI scores of the control group were observed, and skin lesions of the model group increased gradually after administration of IMQ, while mice treated with MTX displayed sparser scales, more shallow erythema, and reduced thickening. Specifically, the each PASI score of the control group was 0, while the PASI score of IMQ-induced psoriasis mouse group increased on the third day due to severe inflammation, and the index of thickness, erythema, and scales increased gradually and reached their peaks on the fifth, sixth, and seventh day, respectively. However, the above three parameters were dramatically improved after treatment with MTX, and the PASI scores were also significantly decreased compared with the IMQ group (Figures 1B–E).

**FIGURE 2:**
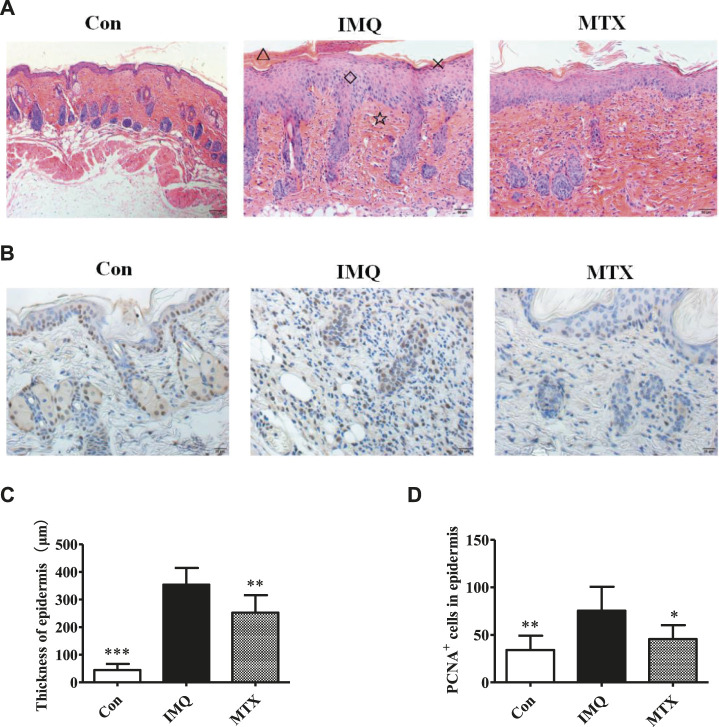
The histological analyses (H&E staining, ×200) of skin lesions and epidermal thickness. △: microlimb swelling; ×: parakeratosis; ◇: acanthosis; ☆: lymphocyte infiltration **(A)**; IHC staining (×200) for proliferating cell nuclear antigen (PCNA) (brown) in mouse back skin **(B)**; epidermal thickness of each group **(C)**; statistical analysis of the number of PCNA^+^ cells in the *epidermis* of each group **(D)**. Data were expressed as mean ± SD (*n* = 8). *p < 0.05, **p < 0.01, and ***p < 0.001, vs. IMQ group.

Pathological changes of skin lesions in the IMQ group, including obvious parakeratosis, acanthosis, and perivascular infiltrations of epithelial inflammatory cells, are similar to the typical phenotype of human psoriatic skin. The thickness of the *epidermis* layer was significantly reduced by MTX treatment, as well as attenuating the IMQ-induced psoriasis (Figure 2A). The vertical *epidermis* thickness of the IMQ group was about eight times greater than that of the control group in microscopy, while MTX treatment also significantly decreased the epidermal thickness (*p* < 0.05) (Figure 2C).

Expression of PCNA is abundant in proliferative cells. PCNA was expressed lower in the skin lesions of the psoriasis mice treated by MTX, indicating that administration of MTX could effectively improve IMQ-induced keratinocyte differentiation and decrease proliferation of keratinocytes triggered by IMQ (Figure 2B). A significant difference (*p* < 0.05) in PCNA levels between MTX and model groups was revealed by image analysis (Figure 2D).

### Serum Metabolomic Profiling of Imiquimod-Induced Psoriasis in Mice and Methotrexate-Treated Psoriasis Mice

#### Multivariate Statistical Analysis

**FIGURE 3:**
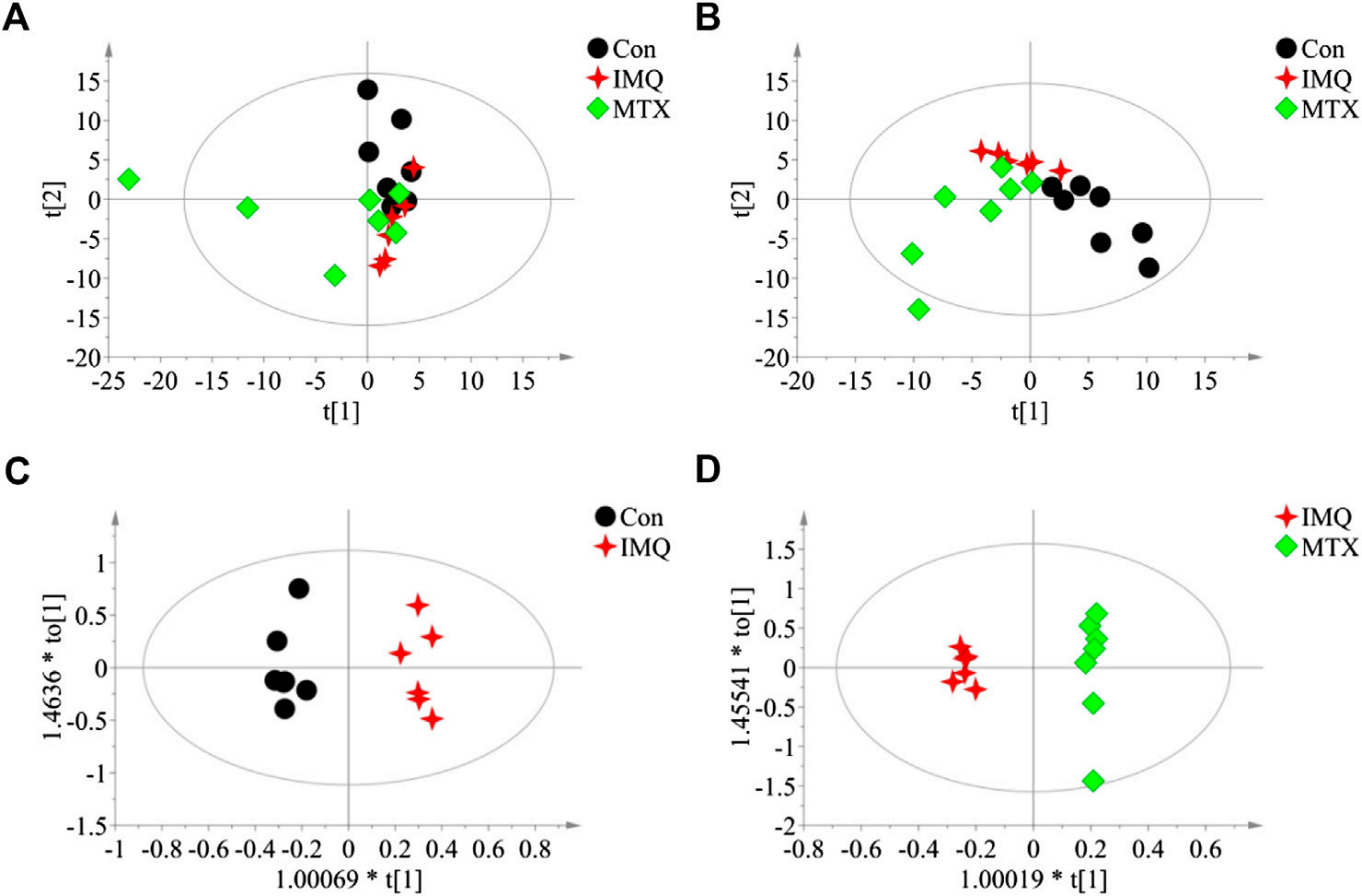
Score plots of multivariate statistical analysis on serum samples (*n* = 7 in the control and MTX groups and *n* = 6 in the IMQ group). **(A)** PCA score plot of control group, IMQ group, and MTX group (R^2^X = 0.812 and Q^2^ = 0.371). **(B)** PLS-DA score plots of control group, IMQ group, and MTX group (R^2^X = 0.823, R^2^Y = 0.947, and Q^2^ = 0.723). **(C,D)** OPLS-DA score plots of serum samples: **(C)** control group vs. IMQ group (R^2^X = 0.97, R^2^Y = 0.993, and Q^2^ = 0.719). **(D)** IMQ group vs. MTX group (R^2^X = 0.826, R2Y = 0.995, and Q^2^ = 0.862). t[1], the first principal component, represents the first largest variance of the data projection, and t[2], the second principal component, represents the second largest variance of the data projection.

Serum metabolome of the psoriasis mice was profiled with metabolomic analysis based on GC/MS. PCA showed a slight separation trend among the three groups (R^2^X = 0.812 and Q^2^ = 0.371; Figure 3A), and PLS-DA score plot displayed a moderate significant metabolic discrimination (R^2^X = 0.823, R^2^Y = 0.947, and Q^2^ = 0.723; Figure 3B), suggesting there was a significant serum metabolic difference among the control, IMQ, and MTX groups. Similarly, the OPLS-DA score plot indicated a very significant profile separation in the control group vs. IMQ group (R^2^X = 0.97, R^2^Y = 0.993, and Q^2^ = 0.719; Figure 3C), and IMQ group vs. MTX group (R^2^X = 0.826, R2Y = 0.995, and Q^2^ = 0.862; Figure 3D). The results illustrated that IMQ-induced psoriasis caused significant alterations of serum metabolites, and one of them was reversed by MTX intervention.

#### Global Metabolomic Alterations of Imiquimod-Induced Psoriasis and Methotrexate Intervention

**FIGURE 4:**
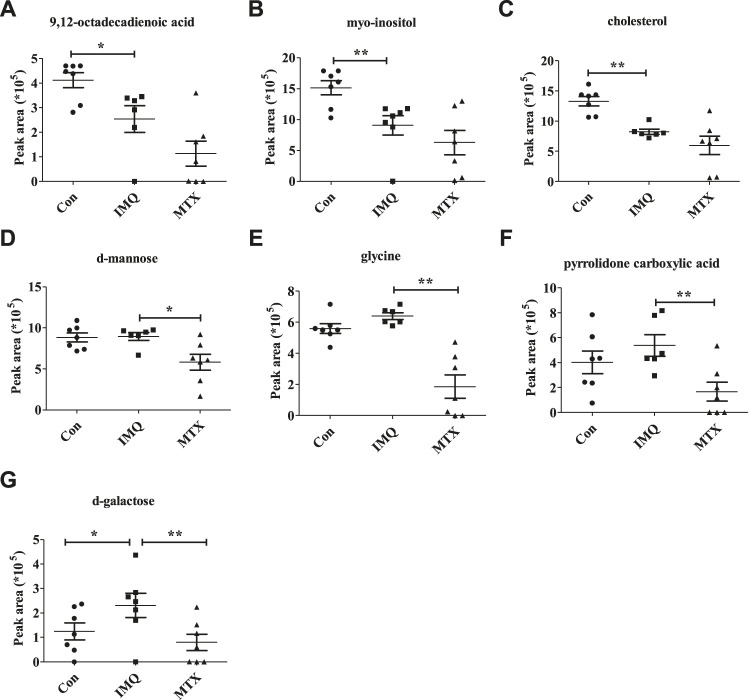
Venn diagrams **(A)** and heat maps **(B)** of identified differential metabolites among the control group, IMQ group, and MTX group.

**FIGURE 5:**
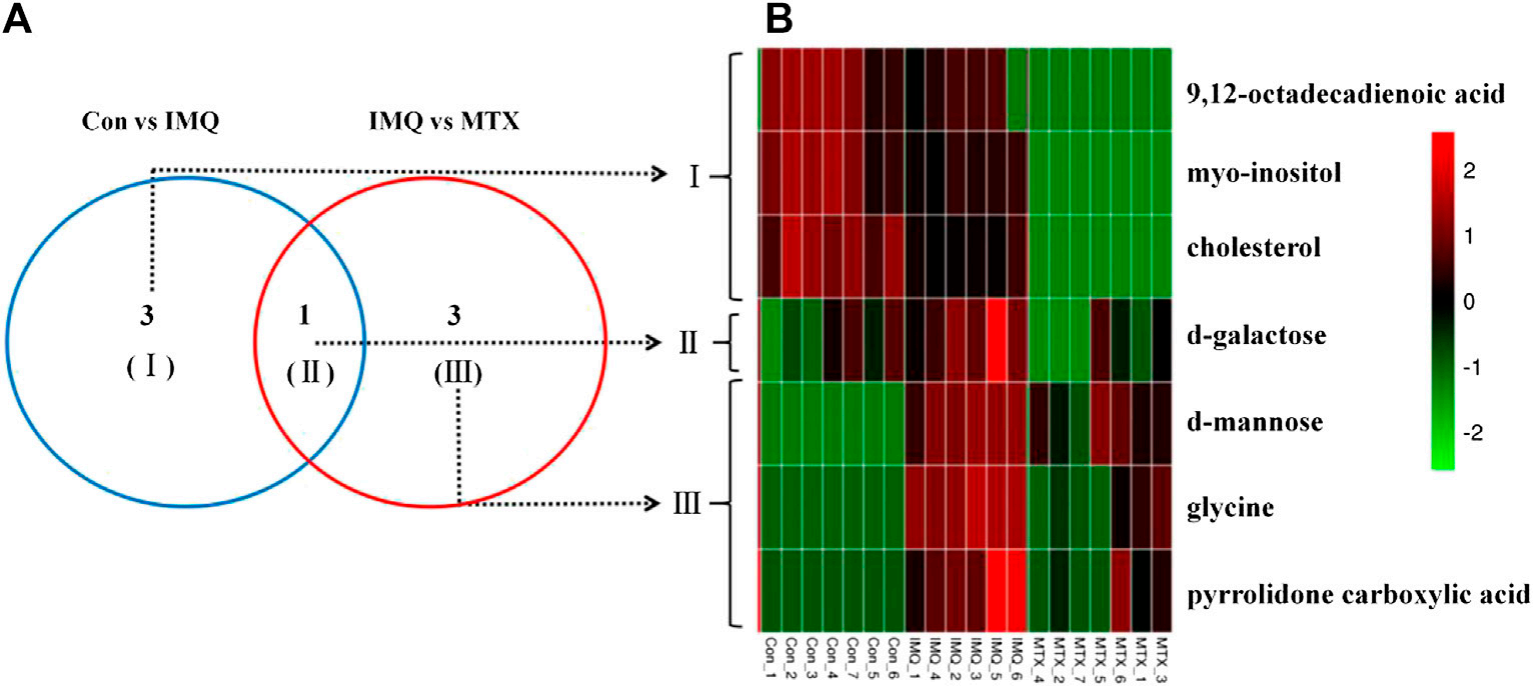
Potential metabolite changes in IMQ-induced psoriasis treated by MTX. Peak area of key metabolites **(A)** 9,12-octadecadienoic acid, **(B)** myo-inositol, **(C)** cholesterol, **(D)** d-mannose, **(E)** glycine, **(F)** pyrrolidone carboxylic acid, and **(G)** d-galactose. **p < 0.01, *p < 0.01, and *p* < 0.05 was considered statistically significant.

A total of seven differential metabolites were identified in the control and MTX groups compared with the IMQ group in the OPLS-DA model and *t*-test differentiation. Among these differential metabolites (VIP > 1 and *p* < 0.05), three of them were present only in the control group, one in both control and MTX groups, and three in the MTX group, which were defined as significantly altered substances for further analysis (Figure 4A). The variations of these differential metabolites were visualized with heat maps described with three clusters (Figure 4B). Cluster I only depicted the metabolic disturbance that appeared in the control and IMQ group. All of them were significantly downregulated such as myo-inositol, 9,12-octadecadienoic acid (Z,Z), and cholesterol. d-Galactose was the upregulated metabolite in cluster II. Three metabolites in cluster III were significantly decreased including glycine, pyrrolidone carboxylic acid, and d-mannose in the MTX group. Myo-inositol cholesterol and 9,12-octadecadienoic acid (Z,Z) in the MTX group were decreased but showed no statistical significance with univariate statistics. In summary, the alteration of the metabolites in cluster I–III indicated the metabolic changes induced by IMQ intervention and MTX treatment, and detailed description of these metabolites is presented in Figure 5 and Table 1.

**TABLE 1:** Differential metabolites among the control group, IMQ group, and MTX group.

Clusters	Metabolites	RT (min)	Mass spectral similarity	*m/z*	Fold change^a^ (vs. IMQ)		*p* value (vs. IMQ)		VIP value^b^ (vs. IMQ)		Metabolic pathways
					Control	MTX	Control	MTX	Control	MTX	
Ⅰ	Myo-inositol	29.16	72	313.39	1.43	1.44	0.01	0.08	1.95	1.42	Inositol phosphate metabolism
	9,12-Octadecadienoic acid (Z,Z)	32.23	97	339.40	1.62	1.93	0.02	0.08	1.06	0.75	—
	Cholesterol	42.00	99	129.10	1.61	1.20	0.00	0.21	2.01	—	Others
Ⅱ	d-Galactose	25.97	70	179.17	0.46	3.23	0.02	0.01	1.01	1.45	Others
Ⅲ	Glycine	12.51	91	248.20	1.02	3.45	0.07	0.00	0.80	1.79	Glyoxylate and dicarboxylate metabolism Glycine, serine, and threonine metabolism
	Pyrrolidone carboxylic acid	17.90	87	156.17	0.87	3.23	0.31	0.01	0.84	1.45	—
	d-Mannose	26.20	91	321.30	1.15	1.54	0.88	0.02	0.01	1.29	Others

IMQ, imiquimod; MTX, methotrexate. *n* = 7 in control and MTX groups, and *n* = 6 in the IMQ group.

a Fold change was calculated as the ratio of the average relative level between the two groups (FC value = control/IMQ or IMQ/MTX).

b VIP value was obtained from OPLS-DA—not detected.

### Metabolic Pathway Analysis

Subsequently, the most relevant pathways were revealed by the analysis of functional pathways promoting beyond the biological interpretation, as shown in Supplementary Figure S2.

KEGG and HMDB were employed to link seven differential metabolites of serum to latent relevant pathways, and the influence values of related pathways were screened via MetaboAnalyst 3.0. The outcomes displayed that two primary metabolic pathways, i.e., inositol phosphate metabolism and galactose metabolism, were affected (*p* < 0.05 or impact factor > 0.1) (Supplementary Figure S2A). On the other hand, glyoxylate and dicarboxylate metabolism; glycine, serine, and threonine metabolism; glutathione metabolism; and galactose metabolism were involved in the MTX group (Supplementary Figure S2B).

## Discussion

In the present research, we established a psoriasis mouse model to investigate the effect of MTX and found that IMQ-induced psoriasis-like skin lesion was significantly ameliorated by MTX. Besides, an integrated metabolomic profiling method based on GC-MS detection was used on IMQ-induced psoriasis mouse model, as well as the treatment with MTX to explore the mechanism of psoriasis and the therapeutic mechanism of MTX on the psoriasis mouse model induced by IMQ.

### Biochemical Interpretation in the Imiquimod Group

#### Disturbed 9,12-Octadecadienoic Acid Metabolism

It was reported that bioactive lipid mediators are important compounds in the occurrence and resolution of inflammation. Of note, there are various locations of targeted lipid mediators in skin and blood, which are not to be influenced by given systemic treatment. The free and total forms of hydroxyoctadecadienoic acid (HODE) derived from linoleic acid have been found to be significantly increased on psoriasis skin lesions. However, it was decreased in serum. Hydroxy fatty acids such as 9- and 13-HODE were found increased significance in psoriasis skin lesions and were produced by the metabolism of omega-6 polyunsaturated fatty acids by lipoxygenase (LOX) 5-LOX, 12-LOX, and 15-LOX (Sorokin et al., 2018). Moreover, 9-HODE promoted the production of various inflammatory cytokines. (Hattori et al., 2008). 9-HODE was found to have proatherogenic properties (Jira et al., 1998), and atherosclerosis is a disease closely related to psoriasis (Siddiqi and Ridker, 2018; Mehta et al., 2012). Similar to the finding (Wang et al., 2017), 13-HODE may be a mechanistic link between psoriasis and cardiovascular complications associated with psoriasis. In addition, supplementations, i.e., omega-3 docosapentaenoic acid and eicosapentaenoic acid, could reduce 9- and 13-HODE and their oxidation products (Markworth et al., 2016). Interestingly, our research showed that 9,12-octadecadienoic acid was also reduced in the serum of the psoriasis group.

#### Disturbed Cholesterol Metabolism

Previous study has reported that psoriasis patients were accompanied by abnormal bile acid function, which is manifested by the lower levels of glycocholate, the combined primary bile acids, glycochenodeoxycholate, as well as secondary bile acids. These alterations lead to plasma cholesterol precursor in the psoriasis group significantly increased (Sorokin et al., 2018). However, it was reported that the application of IMQ in mice led to dietary reduction and weight loss and subsequently drops in lipid levels, especially TC and LDL (Xie et al., 2017), which is very similar to our results. We found the body weights of mice in the procedure of IMQ application in our study were also reduced, and cholesterol in our metabolomics results was significant reduced (VIP score >1 and *p* value <0.05). This difference indicates that there may be significant differences between IMQ-induced psoriasis mouse model and clinic psoriasis patients.

#### Disturbed d-Galactose and Myo-Inositol Metabolism

Exposure to d-galactose can cause a series of age-related diseases, which are considered initiated by the formation of advanced glycation end products (Zhang et al., 2013). As the result of d-galactose treatment, the AGE content in the circulation of mice increased, and this further caused oxidative stress and increased inflammation. In addition, excessive d-galactose caused the overproduction of reactive oxygen species (ROS), which causes increased malondialdehyde (MDA) content and inflammatory cytokines (tumor necrosis factor (TNF-α), interleukin 1β (IL-1β), and IL-6), as well as activities of superoxide dismutase (SOD), glutathione peroxidase (GSH-Px), catalase (CAT), and glutathione content in circulation (Wang et al., 2020). These accompanying results of abnormal d-galacotose metabolism are in accordance with the pathogenesis of psoriasis (Kopeć-Pyciarz et al., 2018).

Inositol depletion is the functional basis of lithium in bipolar affective disorders. Related literature proposed that the inositol supplements had a remarkably advantageous effect on psoriasis patients taking lithium (Allan et al., 2004). Combined with our research, it is speculated that the reduction of inositol levels in psoriasis mice may be one of the mechanisms of psoriasis. Moreover, the results of IMQ-induced redcution of inositol in psoriasis mice are consistent with published literature (Sitter et al., 2013).

### Biochemical Interpretation in the Methotrexate Group

#### Disturbed d-Galactose, d-Mannose, and Glycine Metabolism

MTX, which is one of the most common first-line therapies used for patients with psoriasis (Coates and Helliwell, 2016; West et al., 2017), is a folic acid analog that prevents DNA synthesis by obstructing the biosynthesis of thymidine and purine. Efficient keratinocyte proliferation is a typical feature of psoriasis, which required the synthesis of more protein completed by augmenting amino acids to meet the demand of excessive cell growth (de Koning et al., 2012). Furthermore, keratinocytes can use galactose, fructose, and fatty acids as metabolic substrates to facilitate growth in the absence of glucose transporter type 1 (GLUT1, Zhang et al., 2018). In the present study, the levels of d-glycine, d-galactose, and mannose in MTX-treated psoriasis mice were reduced, which might be explained by MTX-lowered requirements for sugars and amino acids uptake of controlled keratinocytes via target-enhancing biosynthetic requirements of proliferating cells, as shown in Table 1. As the discussion above, increased d-galactose might be the pathogenesis of IMQ-induced psoriasis, and the reduction in the level of d-galactose in serum caused by MTX treatment could be considered as one of its mechanisms on psoriasis. It was reported that mannose can be used to modify the surface of acid-impressible sheddable PEGylated nanoparticles, which not only can rapidly deliver the anti-inflammatory agent to the chronic inflammation sites but also can further expand their distribution and increase their residence time in the mouse model (O’Mary et al., 2017). Similar pieces of literature also reported the relationship between mannose and inflammation (Lossio et al., 2017; Gan et al., 2018).

On the other hand, we speculate that altered galactose, mannose, and glycine levels in the MTX group may be related to the inhibition of glucose transporter and the synthesis of glutathione with anti-inflammatory effects. In addition to glucose, GLUT1 possesses transport activity for mannose and galactose (Zhao, 2014) and controls the epidermal proliferation and acanthosis through chemical inhibition. The deletion of the GLUT1 gene can rescue epidermal acanthosis and proliferation locally. Furthermore, in animal or organoid models of psoriasis, chemical inhibition on GLUT can rescue the proliferation of keratinocytes and prevent inflammation (Zhang et al., 2018). Glutathione is an important scavenging antioxidant, which is opposite to the proinflammatory signaling of hydrogen peroxide. Glutathione is composed of glutamic acid, cysteine, and glycine. Studies also found that dietary glycine supplements can also regulate glutathione synthesis (Atkuri et al., 2007; Dodd et al., 2008). Moreover, an augment in glycine intake could be expected to further promote glutathione synthesis under certain given conditions (McCarty et al., 2018).

#### Disturbed Pyrrolidone Carboxylic Acid Metabolism

GC-MS detection confirmed the presence of 2-pyrrolidone-5-carboxylic acid in human *epidermis*, and compared with the normal horny outer layer of skin, the content of pyroglutamate in the skin scales of psoriatis is much lower (Marstein et al., 1973). It has been proven that most or all of the pyrrolidone carboxylic acid in the horny outer layer of the mammalian skin is derived from histidine-rich proteins of the keratohyalin particles (Scott et al., 1982). Microinflammation could decrease pyrrolidone carboxylic acid production, and the level of pyrrolidone carboxylic acid in dry skin of atopic dermatitis lesions may reflect the clinical severity of atopic dermatitis patients, as well as skin barrier action and lesional inflammation (Jung et al., 2014). It has been known that phytosphingosine has anti-inflammatory and antibacterial functions and induces epidermal differentiation. The phytosphingosine effect on filaggrin metabolism was manifested by increased skin hydration and pyrrolidone carboxylic acid of human skin *in vivo* (Choi et al., 2017). Regarding improvements in psoriasis measurement, there was a significant decrease in pyrrolidone carboxylic acid as shown in Table 1. We could infer from our result that there was a significant difference between animal models and clinical samples, and another reasonable explanation may be that MTX treatment of psoriasis mice was not enough to get the content of pyrrolidone carboxylic acid back to the normal level or MTX works by lowering pyrrolidone carboxylic acid.

The above features implied that MTX can be used to treat inflammatory and hyperplastic diseases (Figure 1). Besides, this phenomenon was identical of what we expected and was consistent with previous research (Zhao et al., 2016; Meng et al., 2017). Furthermore, metabolomics approach was combined to clarify the metabolic alterations and observe potential biomarkers in IMQ-induced psoriasis mice, as well as with MTX treatment. As previously discussed, the development of psoriasis was closely linked to four metabolic changes, i.e., d-galactose, myo-inositol, 9,12-octadecadienoic acid, and cholesterol (*p* < 0.05 and VIP > 1). In addition, four differential metabolites, i.e., glycine, PCA, d-galactose, and d-mannose, had different levels of correlation with the therapeutic effect of MTX on psoriasis mice (*p* <0.05 and VIP >1).

## Conclusion

In conclusion, these results suggest that the changes in serum metabolic phenotype are related to the development of psoriasis and the treatment of MTX. Some potential metabolites and their pathways may become useful clues and research focuses in the future, especially, the effects of d-galactose in psoriasis and MTX treatment are worthy of further study. However, we have to acknowledge that there are certain limitations in our study. It only studied one time-point of psoriasis model and cannot reflect the dynamic procedure of psoriasis and the treatment of MTX. A larger size of sample is required to verify our conclusions. As there are differences between animal models and clinical diseases, further clinical translational studies are urgently needed.

## Data Availability Statement

The raw data supporting the conclusions of this article will be made available by the authors, without undue reservation.

## Ethics Statement

The animal study was reviewed and approved by the Shanghai University of Traditional Chinese Medicine.

## Author Contributions

JZ, JC, YF, and JS performed the research, analyzed the data, and wrote the manuscript. TZ, MZ, and WP contributed to experimental conception and design. JZ, YF, and WP helped in animal experiments. JZ, JC, and JS administered metabolomics studies and interpreted the data. LY, TZ, and MZ revised the manuscript. LY, TZ, and MZ designed and funded the research and finally approved the submission of this manuscript.

## Funding

This project was supported by Program of Shanghai Academic/Technology Research Leader (17XD1403500), In-budget Project from Shanghai University of Traditional Chinese Medicine (Grant no. 18LK006), Shanghai Municipal Education Committee (Grant no. 2013JW17), and Innovation Project for Undergraduates of Shanghai University of Traditional Chinese Medicine (Grant no. 2019SHUTCM183).

## Conflict of Interest

The authors declare that this study was implemented without any commercial or financial relationships that could be interpreted as a potential conflict of interest.
