# Supplementary material for: Serum Metabolomic Profiling Reveals the Amelioration Effect of Methotrexate on Imiquimod-Induced Psoriasis in Mouse
Source: Front Pharmacol. 2020 Nov 19;11:558629. doi: 10.3389/fphar.2020.558629 (PMC7751755; doi:10.3389/fphar.2020.558629)
Supplement: Supplementary file 1 [file datasheet1.docx]

**Serum Metabolomic Profiling Reveals the Amelioration Effect of Methotrexate on Imiquimod-induced Psoriasis in Mouse**

Jiaxin Zong^1,#^, Jieyi Cheng^2,3#^, Yuanfeng Fu^1^, Jing Song^2^, Weisong Pan^4^, Li Yang^2^, Ting Zhang^1,*^, Mingmei Zhou^2,*^

**Supplementary Information**

**Table S1**. The mass spectral similarity of substances at the same retention time as the differential metabolites

| Differential metabolites | Rank | Name | Formula | Mass Spectral Similarity |
| --- | --- | --- | --- | --- |
| myo-inositol | 1 | myo-inositol | C_6_H_12_O_6_ | 72 |
|  | 2 | inositol | C_6_H_12_O_6_ | 43 |
|  | 3 | arabinose | C_5_H_10_O_5_ | 22 |
|  | 4 | .beta-d-galactofuranose | C_16_H_22_O_11_ | 10 |
|  | 5 | neo-inositol | C_6_H_12_O_6_ | 10 |
|  | 6 | d-arabinose | C_5_H_10_O_5_ | 10 |
|  | 7 | .beta.-L-arabinopyranose | C_5_H_10_O_5_ | 9 |
|  | 8 | arabinopyranose | C_5_H_10_O_5_ | 9 |
| 9,12-octadecadienoic acid (Z,Z) | 1 | 9,12-octadecadienoic acid (Z,Z) | C_18_H_32_O_2_ | 97 |
|  | 2 | silane | H_4_Si | 22 |
|  | 3 | linolenic acid | C_18_H_30_O_2_ | 15 |
|  | 4 | propanoic acid | C_3_H_6_O_2_ | 10 |
|  | 5 | acetic acid | C_2_H_4_O_2_ | 10 |
|  | 6 | 9H-fluorene-2-carboxylic acid | C_14_H_10_O_2_ | 10 |
|  |  | methadone N-Oxide | C_21_H_27_NO_2_ | 9 |
| cholesterol | 1 | cholesterol | C_27_H_46_O | 99 |
|  | 2 | 3.beta.-(trimethylsiloxy)cholest-4-ene | C_30_H_54_OSi | 91 |
|  | 3 | cholestane | C_27_H_48_ | 91 |
|  | 4 | cholesterol | C_27_H_46_O | 76 |
|  | 5 | cholesta-2,4-diene | C_27_H_44_ | 60 |
|  | 6 | cholesta-3,5-diene | C_27_H_44_ | 46 |
|  | 7 | cholesta-7,14-diene | C_27_H_44_ | 43 |
|  | 8 | cholesteryl valerate | C_32_H_54_O_2_ | 35 |
|  | 9 | cobaltocene | C_10_H_10_Co | 35 |
|  | 10 | cholest-5-en-3-ol (3.beta.)-, propanoate | C_30_H_50_O_2_ | 20 |
|  | 11 | cholest-5-en-3-ol (3.beta.)-, 9-octadecenoate, (Z) | C_45_H_78_O_2_ | 20 |
|  | 12 | cholest-5-en-3-ol (3.beta.)-, 3,5-dinitrobenzoate | C_34_H_48_N_2_O_6_ | 20 |
|  | 13 | 5-cholesten-3beta-yl isobutyl carbonate | C_32_H_54_O_3_ | 18 |
|  | 14 | ibogamine-18-carboxylic acid, 12-methoxy-, methyl ester | C_22_H_28_N_2_O_3_ | 18 |
|  | 15 | cholest-5-en-3-ol, (3.alpha.)- | C_27_H_46_O | 18 |
| d-galactose | 1 | d-galactose | C_6_H_12_O_6_ | 70 |
|  | 2 | d-mannose | C_6_H_12_O_6_ | 70 |
|  | 3 | d-glucose | C_6_H_12_O_6_ | 70 |
|  | 4 | D-mannitol | C_6_H_14_O_6_ | 58 |
|  | 5 | benzoic acid | C_7_H_6_O_2_ | 14 |
|  | 6 | ribitol | C_5_H_12_O_5_ | 10 |
|  | 7 | 4-quinolinecarboxylic acid | C_10_H_7_NO_2_ | 10 |
| glycine | 1 | glycine | C_2_H_5_NO_2_ | 91 |
|  | 2 | undecane | C_11_H_24_ | 46 |
|  | 3 | .delta. amino levulinic acid | C_5_H_9_NO_3_ | 43 |
|  | 4 | cadaverine | C_5_H_14_N_2_ | 40 |
|  | 5 | 1,4-butanediamine | C_4_H_12_N_2_ | 40 |
|  | 6 | ethylenediamine | C_2_H_8_N_2_ | 38 |
|  | 7 | diethyl aminomalonate | C_7_H_13_NO_4_ | 32 |
|  | 8 | phenylethanolamine | C_8_H_11_NO | 10 |
|  | 9 | pyrimidine | C_4_H_4_N_2_ | 9 |
|  | 10 | 3-chloro-6-hydroxy-4-pyridazinecarboxylic acid | C_5_H_3_ClN_2_O_3_ | 9 |
| pyrrolidone carboxylic acid | 1 | pyrrolidone carboxylic acid | C_5_H_7_NO_3_ | 87 |
|  | 2 | L-proline | C_5_H_9_NO_2_ | 80 |
|  | 3 | 2-piperidinecarboxylic acid | C_6_H_11_NO_2_ | 59 |
|  | 4 | cyclopentanecarboxylic acid | C_6_H_10_O_2_ | 38 |
|  | 5 | d-glucitol | C_6_H_14_O_6_ | 25 |
|  | 6 | acetic acid | C_2_H_4_O_2_ | 9 |
|  | 7 | propanoic acid | C_3_H_6_O_2_ | 9 |
|  | 8 | tebuthiuron | C_9_H_16_N_4_OS | 9 |
| d-mannose | 1 | d-mannose | C_6_H_12_O_6_ | 91 |
|  | 2 | d-glucose | C_6_H_12_O_6_ | 91 |
|  | 3 | D-mannitol | C_6_H_14_O_6_ | 64 |
|  | 4 | sedoheptulose | C_7_H_14_O_7_ | 58 |
|  | 5 | d-galactose | C_6_H_12_O_6_ | 53 |
|  | 6 | glucose | C_6_H_12_O_6_ | 37 |
|  | 7 | glucitol | C_6_H_14_O_6_ | 12 |
|  | 8 | 5-[1,2]dithiolan-4-ylpentanamide | C_8_H_15_NOS_2_ | 10 |
|  | 9 | isoquinoline | C_9_H_7_N | 10 |
|  | 10 | pyrimidine | C_4_H_4_N_2_ | 10 |


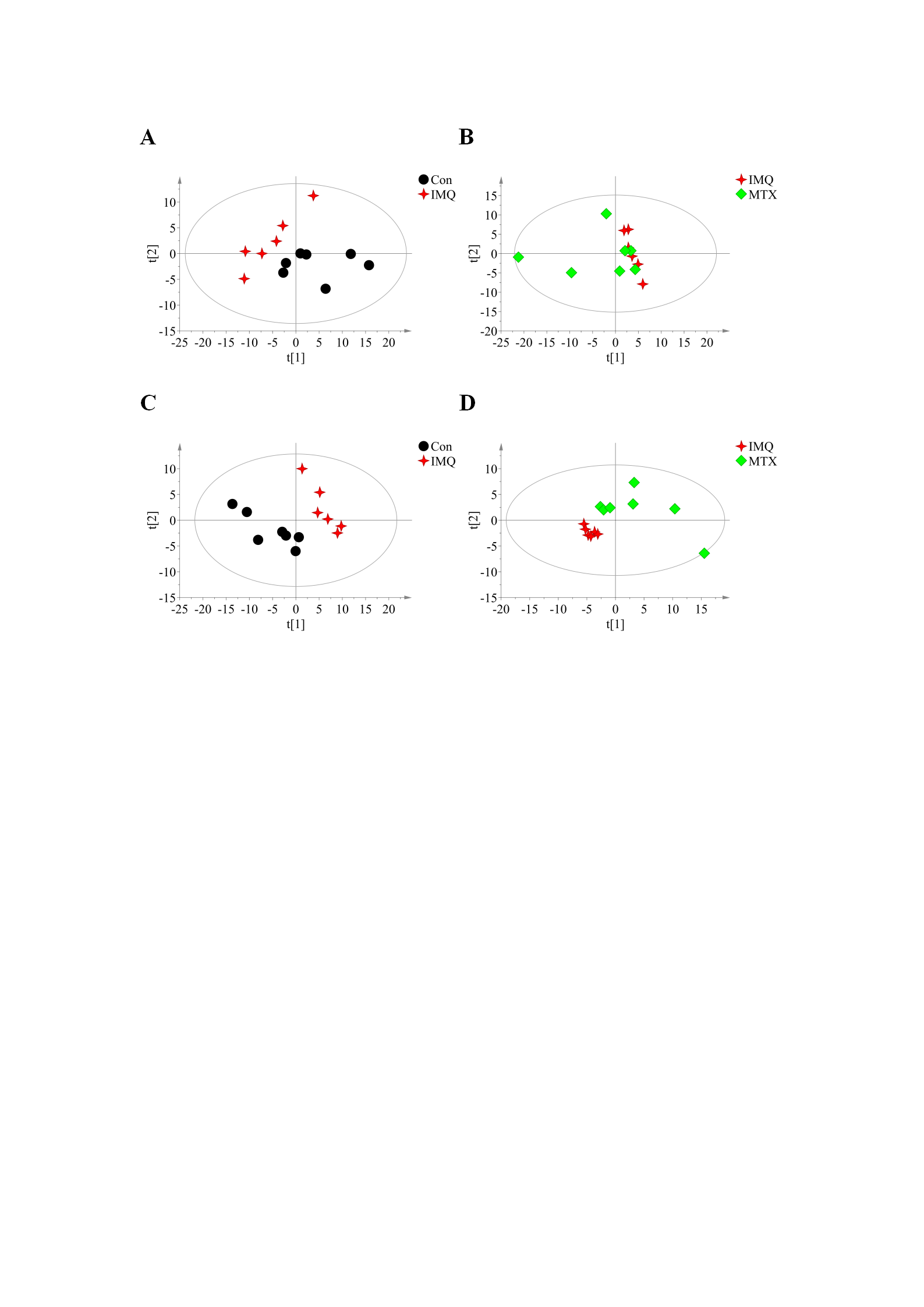


**Figure S1.** Score plots of multivariate statistical analysis on serum samples (n = 7 in the group of Con and MTX, n = 6 in the group of IMQ). (**A** and **B**) PCA scores plots of serum samples. (**A**) Con group versus IMQ group (R^2^X=0.787, Q^2^=0.413); (**B**) IMQ group versus MTX group (R^2^X=0.811, Q^2^=0.334). (**C** and **D**) PLS-DA scores plots of serum samples. (**C**) Con group versus IMQ group (R^2^X=0.713, R^2^Y=0.969, Q^2^=0.883); (**D**) IMQ group versus MTX group (R^2^X=0.826, R2Y=0.995, Q^2^=0.862). t[1], the first principal component, represents the first largest variance of the data projection; t[2], the second principal component, represents the second largest variance of the data projection.


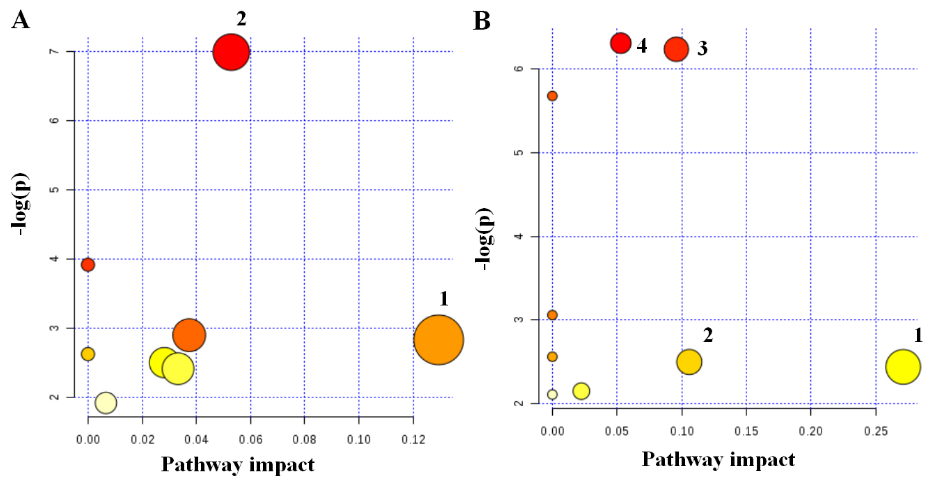


**Figure S2.** Pathway analysis of serum differential metabolites using Metaboanalyst 3.0. **(A)** The metabolic pathway analyzed from the serum differential metabolites between the Con and IMQ group: (1) inositol phosphate metabolism; (2) galactose metabolism. **(B)** The metabolic pathway analyzed from the serum differential metabolites between the IMQ and MTX group: (1) glycine, serine and threonine metabolism; (2) glyoxylate and dicarboxylate metabolism; (3) glutathione metabolism; (4) galactose metabolism.
